# Biomechanical and histomorphological analysis of the mandible in rats with chronic kidney disease

**DOI:** 10.1038/s41598-023-49152-8

**Published:** 2023-12-11

**Authors:** Ta-la Hu, Jun Chen, Shen-quan Shao, Le-le Li, Can Lai, Wu-niri Gao, Rui-feng Xu, Yan Meng

**Affiliations:** 1 Department of Nephrology, The Affiliated Hospital of Inner Mongolia Medical University, Hohhot, 010050 Inner Mongolia China; 2 School of Life Sciences and Biopharmaceutics, Guangdong Pharmaceutical University, Guangzhou, 510006 Guangdong China; 3 Graduate School, Inner Mongolia Medical University, Hohhot, 010110 Inner Mongolia China

**Keywords:** Diseases, Medical research, Nephrology, Risk factors, Urology

## Abstract

The present study aimed to investigate the biomechanical and histomorphological features of mandibles in an adenine-induced chronic kidney disease–mineral and bone disorder (CKD-MBD) rat model of CKD. A total of 14 Sprague-Dawley rats were randomized into the following two groups: control group and CKD group. At the end of the sixth week, all rats were euthanized, and serum was collected for biochemical marker tests. Macroscopic bone growth and biomechanical parameters were measured in the right hemimandible, while the left hemimandible was used for bone histomorphometric analysis. Compared to the control group, the CKD group showed a significant increase in serum creatinine, blood urea nitrogen, and serum parathyroid hormone at the end of the sixth week. The biomechanical structural properties significantly decreased in the CKD group compared to the control group. Bone histomorphometric analysis indicated that the trabecular bone volume of rats in the CKD group was significantly lower than that of the control group. In the CKD groups, the bone formation parameters of the trabecular bone were significantly increased, while the bone mineralization apposition rates of both the trabecular bone and periosteal cortical bone were significantly increased. The rat CKD model showed deteriorated structural mechanics, low trabecular bone volume, high trabecular bone formation, increased trabecular bone mineralization apposition rate, and increased cortical bone mineralization apposition rate, which met the characteristics of osteitis fibrosa, indicating that this model is a useful tool for the study of mandible diseases in CKD patients.

## Introduction

Chronic kidney disease (CKD) is an abnormality of kidney structure and/or function with a variety of causes that persist for over three months, and it is characterized by high prevalence, persistent existence, slow progression, and poor prognosis^1^. In stages 2 to 5 of CKD, renal osteodystrophy develops in most patients and manifests as osteoporosis, bone pain, and fractures, significantly increasing the patients’ distress and socioeconomic burden. Currently, abnormal mineral metabolism, renal osteodystrophy, and vascular calcification in CKD are collectively referred to as chronic kidney disease–mineral and bone disorder (CKD-MBD)^2^.

Although CKD-MBD is characterized by high prevalence and harmfulness, the understanding of its pathogenesis is limited, and clinical prevention and treatment measures are insufficient, especially for mandible lesions of CKD-MBD. It is estimated that nearly 90% of patients with CKD manifest some symptoms of oral disease, and CKD also causes craniofacial damage. Several studies on mandibular lesions in CKD, including uremic leontiasis ossea, Sagliker syndrome^3,4^, and other mandible abnormalities^5,6^, have been previously reported. Therefore, it is important to strengthen basic research in this area. Load-bearing bones are the most commonly used model to study CKD-MBD. However, the pathology of CKD-MBD-associated mandible lesions remains unclear. At present, animal models of mandibular lesions in CKD-MBD are usually induced by partial renal ablation or autosomal dominant polycystic kidney disease and then evaluated by microcomputed tomography (micro-CT), histological analyses, or biochemical analyses^7–9^. However, these models do not evaluate the mandibles by histomorphometric methods, as suggested by the International Society of Nephrology^10^. In addition, biomechanics is an important evaluation method in the study of metabolic bone disease. This method evaluates bone quality from the perspective of structural mechanics and material mechanics, and the results directly reflect bone quality^11,12^.

We previously developed an adenine-induced renal bone disease model, demonstrating similar clinical manifestations of CKD-MBD, indicating an ideal model to study CKD-MBD^13,14^. In the present study, we evaluated the characteristics of mandibular lesions in the rat CKD model from the perspectives of bone growth, biomechanics, and bone histomorphometry to provide an ideal animal model for the subsequent study of the molecular mechanism of the occurrence and development of CKD mandibular lesions, as well as the development of new drugs and other therapeutic methods.

## Materials and methods

### Materials

Fourteen 13-week-old specific pathogen-free (SPF) male Sprague-Dawley (SD) rats were obtained from the Laboratory Animal Center of Sun Yat-sen University (Guangzhou, China). The animal feed formula was determined by the research group and produced by the Guangdong Medical Laboratory Animal Center (Foshan, China). The feed formula was adjusted from the AIN-93 purified feed formula^15^. The following two customized feeds were used: the first feed had 0.75% adenine, and the second feed had no adenine. Both feeds contained 1.03% phosphorus, 1.11% calcium, and 9% casein.

### Experimental design

The present study was performed in strict accordance with the recommendations of the Guide for the Care and Use of Laboratory Animals of the Ministry of Science and Technology of the People's Republic of China. Each animal was housed individually and kept in a 12-h light/dark cycle at a constant temperature of 22 °C ± 1 °C with a humidity of 50% ± 5%. After adaptation for 2 weeks, 14 male SD rats were divided into the following two groups (n = 7 per group): (1) control group, which was fed an adenine-free diet for 6 weeks; and (2) CKD group, which was fed a 0.75% adenine (China National Pharmaceutical Group Corp., Shanghai) diet for 4 weeks followed by a diet without adenine for 2 weeks. The doses and durations of adenine treatment were determined from the results of a previous study that confirmed mandibular bone deterioration^13,16,17^. Throughout the experimental period, all rats were given free access to water. The two groups of rats were subcutaneously injected with calcein (10 mg/kg) labeling on Days 13, 12, 3, and 2 before necropsy to allow fluorescent labeling of bone tissue for measurement of dynamic parameters^18–20^. At the end of the sixth week, fasting blood was collected for biochemical tests, and all rats were euthanized by exsanguination through the retro-orbital sinus under anesthesia. The right hemimandibles were used for mandibular growth evaluation and biomechanics analysis, and the left hemimandibles were used for bone histomorphometric analysis at necropsy.

### Biochemical markers

Serum creatinine (SCr) and blood urea nitrogen (BUN) were measured using a Hitachi 7600 (HITACHI, Japan). A rat bioactive intact PTH ELISA kit (Immutopics, USA) was used to measure serum parathyroid hormone (PTH) levels.

### Macroscopic growth of the mandible

**Figure 1:**
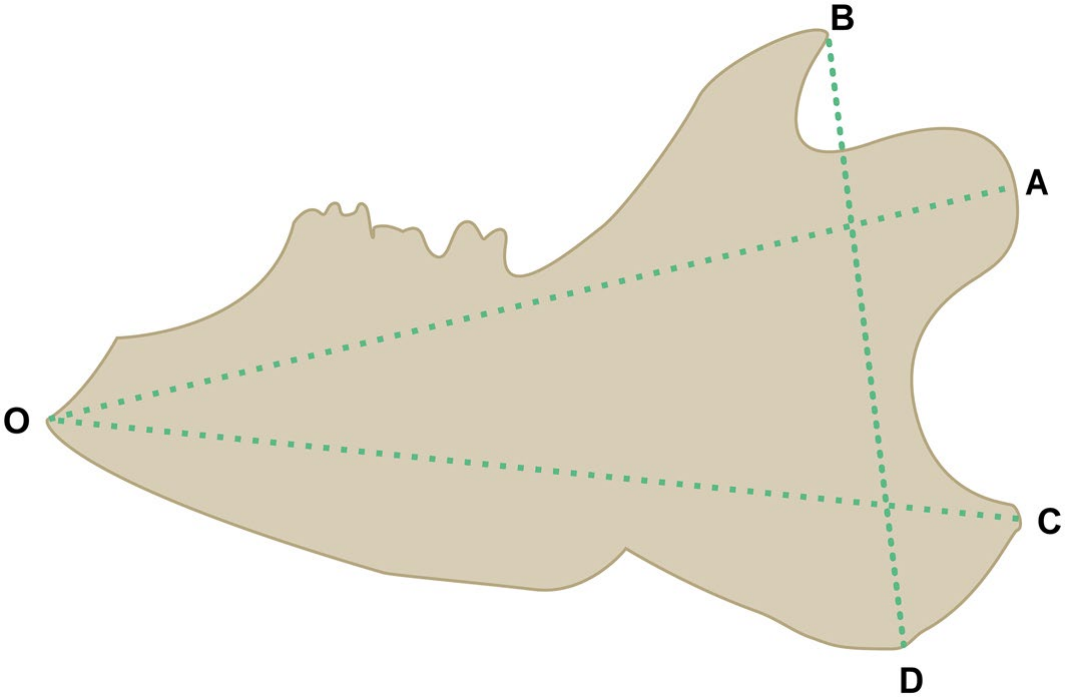
Schematic of the rat mandible showing indices of macroscopic growth measured by digital calipers.

Mandibular growth was measured in the two groups. Measurements between anatomical points with digital calipers were used to estimate growth directly on the right hemimandible (Fig. 1). The mandibular area (S, mm^2^), which corresponds to the size of the bone, was calculated from a triangle formed among three stable points: the most superior posterior point of the coronoid process (B), the most posterior point of the mandible (C), and the most anterior inferior bone point (O). The base of the jaw (length O-C, mm), the mandibular length (length O-A, mm), and the mandibular height (length B-D, mm) were also calculated^21^.

### Biomechanics of the mandible

The right hemimandible underwent a three-point bending mechanical test in the MTS test machine (MTS, USA) after evaluating bone growth to measure biomechanical properties. The laboratory temperature was set to 25 °C, and the relative humidity was 50–60%. Each bone was placed on two lower supports, with the lateral aspect facing down and centered along its length. The supports were equidistant from the bone ends and separated by a constant distance L (distance between supports = 11 mm), equivalent in all cases to no more than two-fifths of the bone length. Loads were applied transversely to the bone axis at a point immediately posterior to the posterior surface of the third molar at a rate of 5.00 mm/min^22–24^. Structural mechanics, including maximum displacement, maximum load, force energy, and stiffness, were then evaluated.

### Mandibular histomorphometry

**Figure 2:**
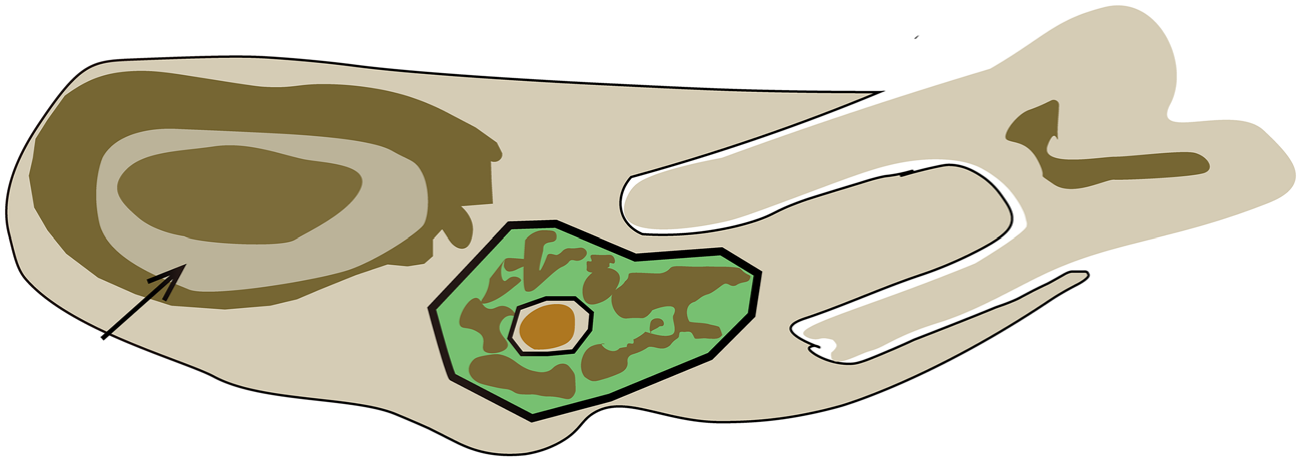
Schematic of the rat mandible showing the regions of interest (ROIs) of the histomorphometric parameters. The green area indicates the ROI of trabecular bone, and the black arrow indicates the ROI of cortical bone, which is the cross-section of the incisor tooth.

The trimmed left hemimandibles were fixed in 4% paraformaldehyde. After dehydrating the bone tissues in ethanol, the tissues were embedded in methyl methacrylate. After full polymerization, frontal cross-sections of the mesial root of the mandibular first molar region were cut (300 μm) and polished (20 μm) with a precision bone saw (cutting grinding system, EXAKT Apparatebau, Norderstedt, Germany)^25^. The unstained sections were used to measure the volume, turnover, and mineralization parameters in the trabecular and cortical bones. Figure 2 shows the regions of interest (ROIs) of the trabecular and cortical bones. The cross-section of the incisor tooth was selected as the cortical bone ROI. The incisor tooth and mandible of rats are conjoined together, indicating the crucial role of the incisor tooth in supporting the mandible. Using the cross-section of the incisor tooth as the cortical bone ROI allowed measurement of the periosteal and endocortical cortical bone parameters at the same time. A semiautomatic image analysis system (Bioquant, Nashville, TN, USA) was utilized to measure the histomorphometric parameters.

The trabecular area (Tb.Ar) was the histomorphometric volume parameter for trabecular bone, and the calculated volume parameters included trabecular thickness (Tb.Th), trabecular number (Tb.N), and trabecular separation (Tb.Sp). The bone formation histomorphometric parameters of the trabecula were as follows: bone formation rate/tissue volume referent (BFR/TV), bone formation rate/bone volume referent (BFR/BV), and bone formation rate/bone surface referent (BFR/BS), which reflects bone turnover with respect to bone formation. The bone mineralization histomorphometric parameter of the trabecula was the mineralization apposition rate (MAR). For cortical bone, the histomorphometric volume parameters were as follows: cortical area (Ct.Ar), percent cortical area (%Ct.Ar), cortical bone area (Ct.B.Ar), marrow area (Ma.Ar), and percent marrow area (%Ma.Ar). The bone formation parameters were as follows: percent periosteal-labeled perimeter (%P-L.Pm), periosteal-BFR/BS referent (P-BFR/BS), percent endocortical-labeled perimeter (%E-L.Pm), and endocortical-BFR/BS referent (E-BFR/BS). The bone mineralization parameters of the cortical bone were periosteal-MAR (P-MAR) and endocortical-MAR (E-MAR). The terminology used in the present study was consistent with the ASBMR Histomorphometry Nomenclature Committee on Standardization of Nomenclature^26^_._

### Statistics

All data were analyzed using SPSS software (version 22.0) and are expressed as the mean ± standard deviation (SD). If the comparison between two groups met the requirements of the parametric test, a two-sample *t* test was used; if the requirement of the parametric test was not satisfied, two independent samples were used for the Mann‒Whitney *U* test. *P* < 0.05 was considered statistically significant for all tests.

### Ethical approval

The present study was approved by the Ethics Committee of the Affiliated Hospital of Inner Mongolia Medical University (No. WZ2021037), and all procedures were conducted in accordance with the Guiding Principles in the Care and Use of Animals (China).

## Results

### Evaluation of the CKD model and biochemical markers of CKD-MBD

To evaluate the status of the CKD model and the biochemical parameters for CKD-MBD, SCr, BUN, and PTH were measured at the end of the sixth week. The CKD model was well established with higher SCr (49.39 ± 11.32 vs. 187.08 ± 47.60 μmol/L, *P* < 0.001), BUN (2.74 ± 0.64 vs. 9.76 ± 3.36 mmol/L, *P* < 0.01), and PTH (475.16 ± 348.25 vs. 4403.79 ± 275.24 pg/l, *P* < 0.001) levels compared to the control group.

### Macroscopic mandibular growth

Mandibular bone growth was evaluated by measuring the right hemimandibles in the control and CKD groups at the end of the sixth week. Although the mean values of the mandibular base (OC, mm), mandibular length (OA, mm), mandibular height (BD, mm), and mandibular area (S, mm^2^) exhibited a decreasing tendency in the CKD group compared to the control group, the difference was not significant (*P* > 0.05).

### Mandibular biomechanical properties

**Figure 3:**
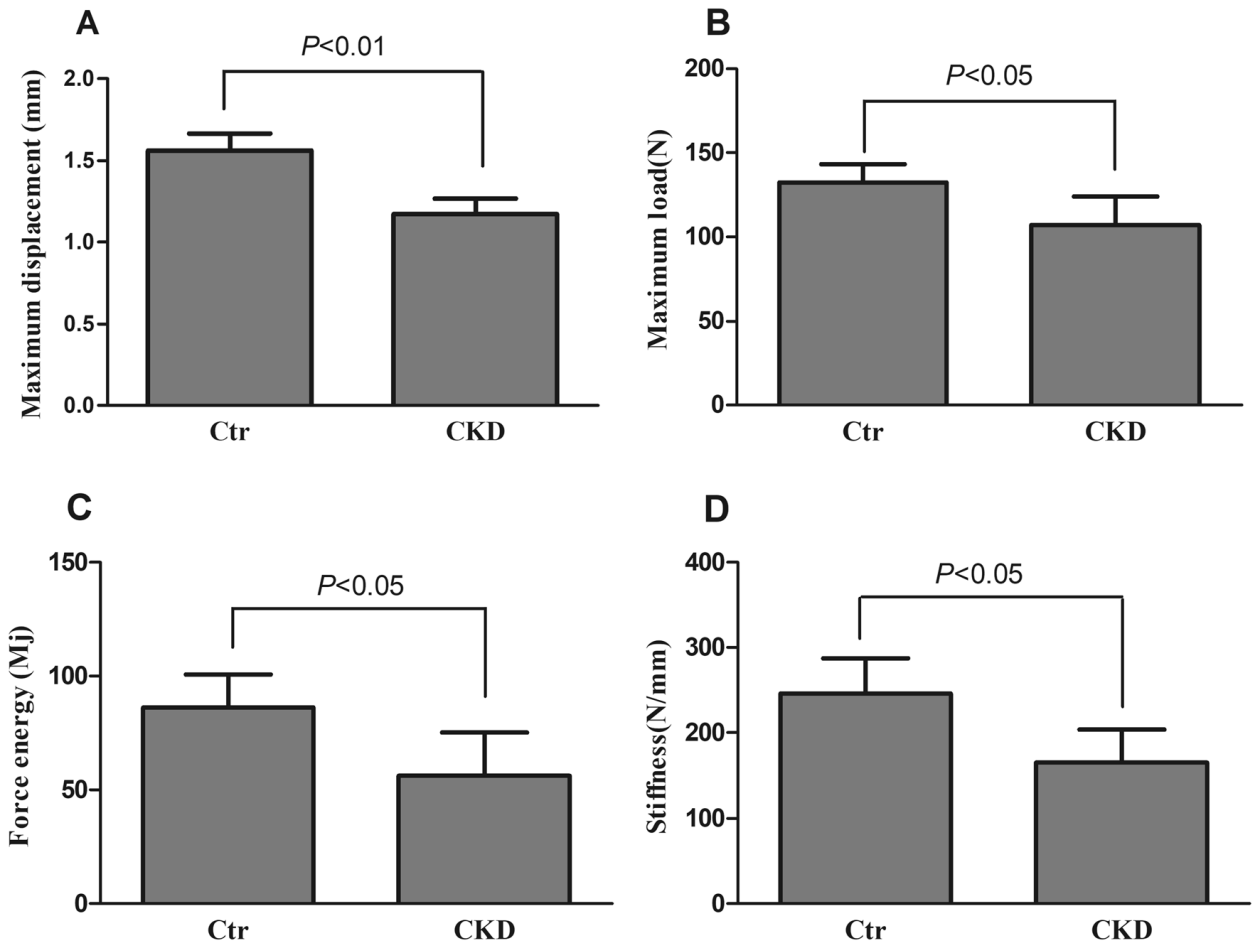
Maximum displacement (**A**), maximum load (**b**), force energy (**C**), and stiffness (**D**) of the right hemimandible in the control and CKD groups at euthanasia. Data are expressed as the mean ± standard deviation (SD) for n = 7 in each group. Ctr, normal control group; CKD, chronic kidney disease group.

Figure 3 shows the structural biomechanical properties of the right hemimandible calculated from the analysis of the load/deformation curve obtained in the mechanical bending test. The maximum displacement, maximum load, load energy, and stiffness were significantly decreased in the CKD group compared to the control group (1.17 ± 0.10 vs. 1.56 ± 0.11 mm, *P* < 0.01; 107.29 ± 16.92 vs. 132.3 ± 10.74 N, *P* < 0.05; 56.17 ± 19.12 vs. 86.18 ± 14.54 Mj, *P* < 0.05; 165.11 ± 39.1 vs. 246.04 ± 40.81 N/mm, *P* < 0.05).

### Mandibular histomorphometry

**Figure 4:**
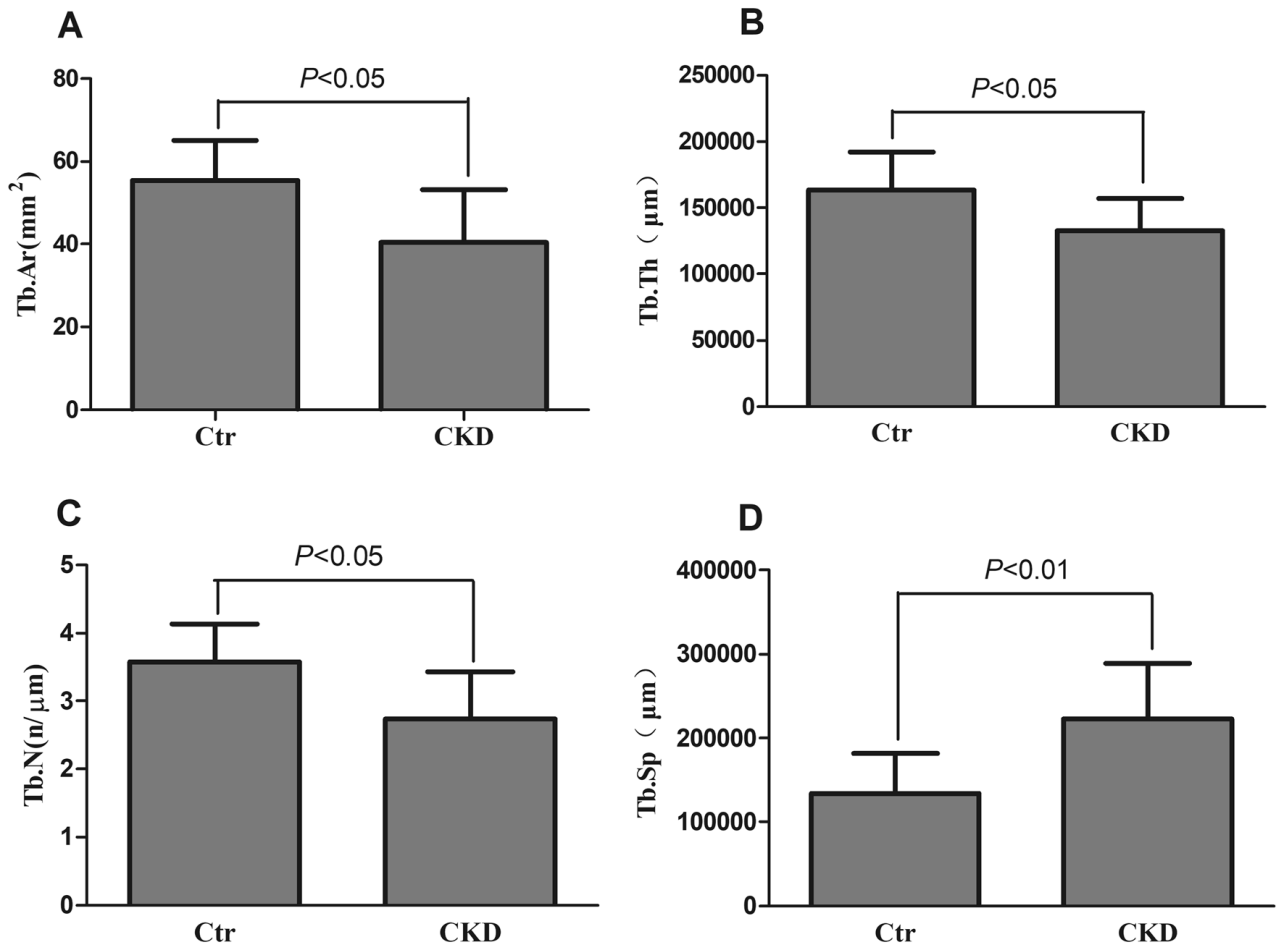
Tb.Ar (**A**), Tb.Th (**B**), Tb.N (**C**), and Tb.Sp (**D**) of the left hemimandible in the control and CKD groups at euthanasia. Data are expressed as the mean ± standard deviation (SD) for n = 7 in each group. Ctr, normal control group; CKD, chronic kidney disease group. Tb.Ar, trabecular area; Tb.Th, trabecular thickness; Tb.N, trabecular number; Tb.Sp, trabecular separation.

**Figure 5:**
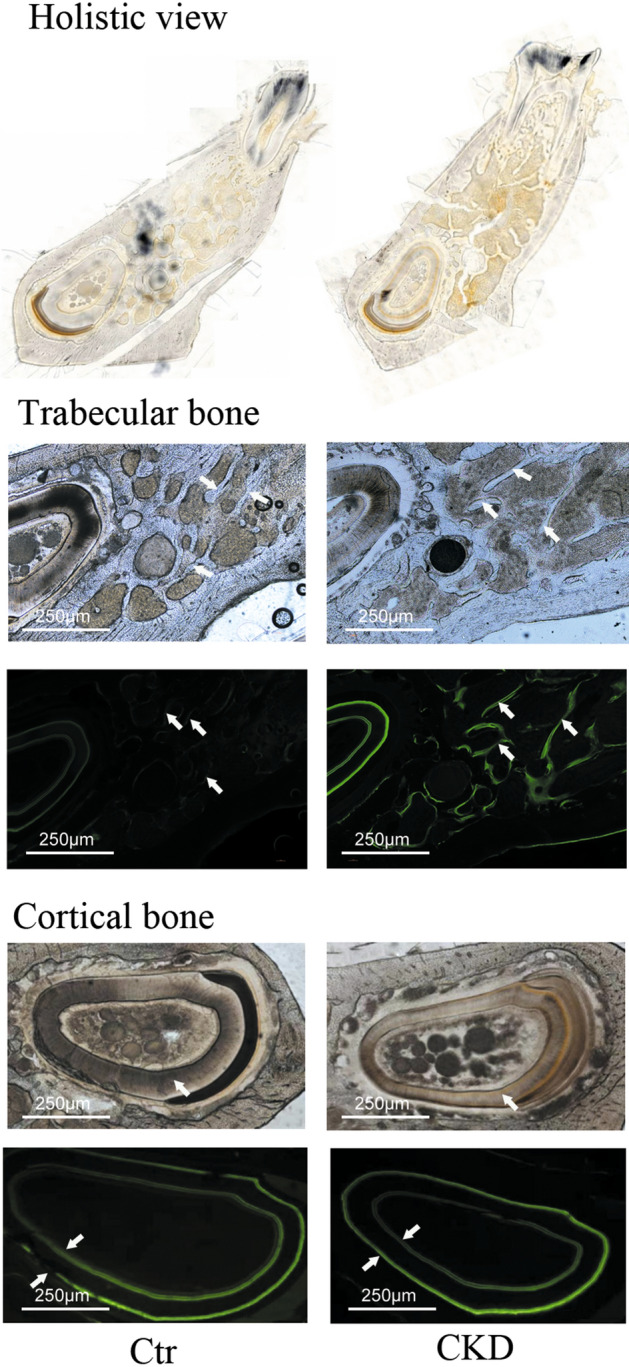
Bone structure and bone formation in the rat mandible in the control and CKD groups. The images in the first line show holistic views of mandible frontal cross-sections without stain in the control and CKD groups (original magnification × 10). The images in the second line show the bone trabeculae of mandible frontal cross-sections without stain in the control and CKD groups (original magnification × 40). The white arrows indicate the bone trabeculae. Compared to the control group, the trabeculae became sparse and slender in the CKD group. The images in the third row show the trabecular fluorescence images of mandible frontal cross-sections without decalcification in the control and CKD groups (original magnification × 40). The fluorescence shows bone formation in the mandible trabeculae (the representative fluorescence is indicated by white arrows), which was labeled by the subcutaneous injection of calcein. Compared to the control group, bright fluorescence indicated active bone formation in the mandible trabeculae in the CKD group. The images in the fourth line show the cortical bone of the mandible frontal cross-section without stain in the control and CKD groups (original magnification × 40), which was the cross-section of the incisor tooth. The white arrows indicate the cortical bone. No significant difference was found between the two groups. The images in the fifth line show the cortical fluorescence images of mandible frontal cross-sections without decalcification in the control and CKD groups (original magnification × 40). The fluorescence shows the bone formation of endocortical and periosteal cortical bones in the mandible (the representative fluorescence is indicated by white arrows), which was labeled by the subcutaneous injection of calcein. Compared to the control group, the fluorescence in the periosteal cortical bone was bright and continuous in the CKD group, which indicated active bone formation. The fluorescence in the endocortical cortical bone showed no difference between the two groups.

The left hemimandible trabecular bone was measured for trabecular bone histomorphometric parameters at the end of the sixth week (Table 1, Figs. 4, and 5). In CKD rats, the values of Tb.Ar, Tb.Th, and Tb.N were significantly decreased, while the value of Tb.Sp was significantly increased. In addition, CKD rats had significantly increased bone formation parameter values, including BFR/TV, BFR/BV, and BFR/BS, compared to control rats. The mineralization-related parameter, MAR, was significantly higher in the CKD group than in the control group.

**Table 1:** Bone histomorphometric results in the trabecular bone at the end of the sixth week.

Group	Ctr	CKD	*P* value
Volume			
Tb.Ar (mm^2^)	55.28 ± 9.74	40.43 ± 12.70^#^	**0.013**
Tb.Th (µm)	163580.00 ± 28629.02	132850.00 ± 24403.91^#^	**0.012**
Tb.N (n/µm)	3.568 ± 0.557	2.739 ± 0.688^#^	**0.013**
Tb.Sp (µm)	133700.00 ± 48387.05	223300.00 ± 65654.92^##^	**0.005**
Turnover			
BFR/TV (%/year)	0.09 ± 0.04	0.19 ± 0.08^##^	**0.001**
BFR/BV (%/year)	0.19 ± 0.10	0.28 ± 0.08^#^	**0.033**
BFR/BS (µm/day × 100)	17.57 ± 7.73	31.01 ± 14.09^#^	**0.010**
Mineralization			
MAR (µm/day)	2.14 ± 0.48	3.11 ± 0.67^##^	**0.001**

Significant values are in bold.

Data are expressed as the mean ± standard deviation (SD) for n = 7 in each group.

Ctr, normal control group; CKD, chronic kidney disease group.

Parameters: Tb.Ar, trabecular area; Tb.Th, trabecular thickness; Tb.N, trabecular number; Tb.Sp, trabecular separation; MAR, mineralization apposition rate; BFR/TV, bone formation rate (tissue area referent); BFR/BV, bone formation rate (bone area referent); BFR/BS, bone formation rate (bone surface referent).

^#^*P* < 0.05 versus the control group.

^##^*P* < 0.01 versus the control group.

The left hemimandible bone was also measured for cortical bone histomorphometric parameters at the end of the sixth week (Table 2 and Fig. 5). The bone volume parameter values (Ct.Ar, %Ct.Ar, Ct.B.Ar, Ma.Ar, and %Ma.Ar) were note significantly different between the two groups. Regarding the periosteal bone formation parameters, %P-L.Pm was significantly increased in the CKD group compared to the control group, and P-BFR/BS demonstrated no significant difference between the two groups. The endocortical bone formation parameters (%E-L.Pm and E-BFR/BS) were not significantly different between the two groups. No significant difference was found in the mineralization-related parameters, P-MAR and E-MAR, between the two groups.

**Table 2:** Bone histomorphometric results in the cortical bone at the end of the sixth week.

Group	Ctr	CKD	*P* value
Volume			
Ct.Ar (mm^2^)	1865700.00 ± 133847.00	1826100.00 ± 295401.00	0.678
%Ct.Ar (%)	66.70 ± 6.56	70.29 ± 12.12	0.133
Ct.B.Ar (mm^2^)	1865700.00 ± 133847.00	1826100.00 ± 295401.00	0.954
Ma.Ar (mm^2^)	940100.00 ± 213244.00	779840.00 ± 338557.00	0.179
%Ma.Ar (%)	33.30 ± 6.56	29.71 ± 12.12	0.133
Turnover			
Periosteal			
%P-L.Pm (%)	73.03 ± 4.04	78.52 ± 5.15^#^	**0.037**
P-BFR/BS (µm/day × 100)	53.69 ± 9.44	60.66 ± 7.88	0.140
Endocortical			
%E-L.Pm (%)	106.40 ± 2.66	101.21 ± 7.40	0.205
E-BFR/BS (µm/day × 100)	96.34 ± 9.91	94.38 ± 18.83	0.768
Mineralization			
Periosteal			
P-MAR (µm/day)	0.68 ± 0.09	0.83 ± 0.12^##^	**0.009**
Endocortical			
E-MAR (µm/day)	0.91 ± 0.10	0.93 ± 0.13	0.729

Significant values are in bold.

Data are expressed as the mean ± standard deviation (SD) for n = 7 in each group.

Ctr, normal control group; CKD, chronic kidney disease group.

Parameters: Ct.Ar, cortical area; %Ct.Ar, percent cortical area; Ct.B.Ar, cortical bone area; Ma.Ar, marrow area; %Ma.Ar, percent marrow area; %P-L.Pm, percent periosteal labeled perimeter; P-MAR, periosteal mineralization apposition rate; P-BFR/BS, periosteal bone formation rate/bone surface referent; %E-L.Pm, percent endocortical-labeled perimeter; E-MAR, endocortical mineralization apposition rate; E-BFR/BS, endocortical bone formation rate/bone surface referent.

^#^*P* < 0.05 versus the control group.

^##^*P* < 0.01 versus the control group.

## Discussion

The earliest pathophysiological changes in CKD can be traced back to the G1 stage. FGF23 is thought to be an early biomarker of disordered phosphorus metabolism in the initial stages of CKD^27^. After CKD progresses to stage G2-3, significant clinical mineral metabolism disorders begin to appear, which manifest as secondary hyperparathyroidism, abnormal bone metabolism, and even life-threatening renal osteodystrophy. Advancements in the knowledge of and therapeutic measures for CKD and its complications, such as secondary hyperparathyroidism (HPT-II) and CKD-MBD, have resulted in a decreased frequency of significant macroscopic bone alterations in kidney patients. Nonetheless, early skeletal change for bone scintigraphy shows increased uptake in the jaws and skull, implying high turnover osteodystrophy^28^. Renal bone disease causes cranial and maxillofacial appearance changes due to maxillary fibrosis deformity. Lopes et al.^29^ reported two cases of severe renal osteodystrophy affecting the maxilla and mandible, ultimately causing facial disfigurement in a young female patient with CKD and a middle-aged female patient with CKD. Severe bone changes in the skull and maxillofacial face are accompanied by abnormal bone metabolism in other sites, which are characterized as Sagliker syndrome^4^. A multicenter retrospective study has reported 4 cases of CKD patients clinically consistent with Sagliker syndrome in a total of 21 patients^30^. Secondary hyperparathyroidism affects up to 92% of patients receiving hemodialysis and may present as a maxillary brown tumor^31^. The majority of macroscopic manifestations of renal osteodystrophy present as diffuse jaw enlargement^6,32^, with the alterations being exclusively in the jaw bones as any manifestation in long bones and aorta were discarded. However, few studies have evaluated CKD mandible lesions in basic research, and few studies have comprehensively evaluated macroscopic bone growth, biomechanics, and histomorphometrics of mandibular lesions in CKD. Therefore, we established adenine-induced CKD-MBD in rats and evaluated the mandibles from the three perspectives mentioned above to explore the pathogenic mechanism of CKD on the mandible.

In the present study, the CKD model was well established and was characterized by higher SCr, BUN, and PTH levels than those in normal rats. The mean values of the mandibular base (OC, mm), mandibular length (OA, mm), mandibular height (BD, mm), and mandibular area (S, mm^2^) did not differ significantly between the two groups. The present results were not consistent with the suppressive effects of corticosteroids on mandibular bone growth^21^ and face and jaw skeletal lesions in dogs with secondary parathyroid hyperplasia^33^. This inconsistency may be due to the different mechanisms of CKD, corticosteroids, and secondary parathyroid hyperplasia in mandibular macroscopic growth.

Bone biomechanics is a branch of biomechanics based on the theory of engineering mechanics. Studying the mechanical properties of bone tissue under external action and the biological effects of bone after stress is a reliable method to assess bone quality, and it is one of the best methods to evaluate various measures against bone loss^34^. The mechanical properties of bone include structural and material mechanical properties. In the present study, we evaluated the structural mechanical properties of the right hemimandibles, which demonstrated significantly decreased structural mechanical properties in the CKD group compared to the control group. These results were consistent with the results of femur and lumbar vertebrae in adenine-induced CKD rats^14,35^. The material mechanical properties are also decreased in the adenine-induced CKD rats. In the present study, we did not measure the material mechanical parameters of the mandible as we were unable to find an appropriate method to measure the irregular bone, but we inferred the material mechanical properties of mandible also deteriorated in CKD. Our future studies are planned to investigate the material mechanical properties.

Bone histomorphometry is a quantitative stereological research technique based on quantitative measurement of bone sections, including cellular and histologic morphological changes. The skeletal lesions of the systemic disorders of CKD-MBD are quantifiable by histomorphometry of bone biopsies. Histologic classification is based on the turnover, mineralization, and volume (TMV), which is a standardized nomenclature for reporting the results of bone histomorphometry for both clinical and research purposes^10^. Turnover reflects the rate of skeletal remodeling, which is normally a coupled process of bone resorption and bone formation. Bone formation rates and activation frequency are acceptable parameters for assessing bone turnover. In the present study, the bone formation parameters in the trabecular bone showed that the CKD group had significantly increased BFR/TV, BFR/BV, and BFR/BS compared to the control group. Bone formation parameters in the periosteal cortical bone showed that the CKD group had significantly increased %P-L.Pm compared to the control group, and the P-BFR/BS showed an increased trend in the CKD group compared to the control group. Tegarding bone formation parameters, there was no significant difference in the endocortical cortical bone in the CKD group compared to the control group. Although the bone resorption parameters were not measured, these results indicated high bone turnover in the CKD group (Oc.S/BS, osteoclast surface per millimeter bone perimeter; N.Oc/BS, osteoclast number per millimeter bone perimeter). Our previous studies of the lesions of load-bearing bone (femur) in the adenine-induced CKD rat model have demonstrated that the trabecular and cortical bones show a high turnover, including high bone formation and high bone absorption^13,36,37^. The same high bone turnover has also been observed in the proximal tibia in the CKD model induced by polycystic kidney disease^38^ and in the distal femur in the adenine-induced CKD model^39^. Mineralization reflects how well bone collagen becomes calcified tissue during the formation phase of skeletal remodeling. Compared to the control group, the MAR and P-MAR were significantly increased in the CKD group, indicating increased mineralization time. The MAR results agreed with previous studies on the femur in adenine-induced CKD rats^13,36,37^, and they were was consistent with previously reported research on the proximal tibia of CKD rats induced by polycystic kidney disease^38^. Bone volume represents the amount of bone per unit volume of tissue. In the present study, the volume of trabecular bone and cortical bone was measured as recommended by TMV classification. CKD significantly increased the value of Tb.Sp but decreased the values of Tb.Ar, Tb.Th, and Tb.N in trabecular bone. The cortical bone volume parameters, including Ct.Ar, %Ct.Ar, Ct.B.Ar, Ma.Ar, and %Ma.Ar were not significantly different between the CKD and control groups. These results agreed with our previous studies in the femur^13,36^ and other studies in the tibia^35^ and femur^39^ in different CKD models.

Sotaro et al.^40^ investigated the effects of CKD on the structural and mechanical properties of maxillary and mandibular bones in a 5/6 nephrectomy model, and they demonstrated that CKD causes an increase in the number of osteoclasts and a decrease in the number of osteocytes in the maxilla. CKD induces a reduced bone volume in the maxilla and mandible, as well as a decreased BMD in the maxilla and a deteriorated mechanical performance in the maxilla. These results were similar to the results of the present study, but a more severe degree of these indicators was found in the present study, which may be due to the different modeling methods. However, the basic characteristics of renal osteodystrophy reflected by the two models were similar. Interestingly, the severity degree of the indicators was more serious in the maxilla than in the mandible. This difference may be due to the mandible being composed of thick cortical bone while the maxilla is mainly composed of trabecular bone, indicating that bone metabolism was not equally affected in these bones^41^.

According to the TMV classification scheme, the mandibular disorders in the present CKD model met the criteria for mild or severe hyperparathyroid-related bone disease (HPT) or osteitis fibrosa (OF) with medium to high turnover and any bone volume depending on the duration of the disease process.

The present study had several limitations. First, osteoblast-related and osteoclast-related indicators were not measured for bone turnover even though both bone formation and bone resorption parameters influence bone turnover. Second, bone metabolism-related serum markers were not monitored, which prevented a comprehensive evaluation of renal osteodystrophy, Thus, further studies are warranted.

In conclusion, the rat CKD model in the present study showed deteriorated structural mechanics, low trabecular bone volume, high trabecular bone formation, increased trabecular bone mineralization apposition rate, and increased cortical bone mineralization apposition rate, which met the characteristics of secondary hyperparathyroid-related bone disease or osteitis fibrosa of load-bearing bones. Thus, the present model will be a useful tool for the study of mandible diseases in CKD patients.

## Data Availability

The datasets used and/or analyzed during the current study are available from the corresponding author upon reasonable request.

## Abbreviations

BFR/TV: Bone formation rate/tissue volume referent
BFR/BV: Bone formation rate/bone volume referent
BFR/BS: Bone formation rate/bone surface referent
BD: Mandibular height
Ctr: Control
CKD: Chronic kidney disease
CKD-MBD: Chronic kidney disease–mineral and bone disorder
Ct.Ar: Cortical area
%Ct.Ar: Percent cortical area
Ct.B.Ar: Cortical bone area
%E-L.Pm: Percent endocortical-labeled perimeter
E-MAR: Endocortical-MAR
E-BFR/BS: Endocortical-BFR/BS referent
MAR: Mineral apposition rate
Ma.Ar: Marrow area
%Ma.Ar: Percent marrow area
N.Oc/BS: Osteoclast number per millimeter bone perimeter
OC: Mandibular base
OA: Mandibular length
Oc.S/BS: Osteoclast surface per millimeter bone perimeter
%P-L.Pm: Percent periosteal-labeled perimeter
P-MAR: Periosteal-MAR
P-BFR/BS: Periosteal-BFR/BS referent
ROIs: Regions of interest
S: Mandible area
SPF: Specific pathogen free
SD: Sprague-Dawley
Tb.Ar: Trabecular area
Tb.Th: Trabecular thickness
Tb.N: Trabecular number
Tb.Sp: Trabecular separation

## References

[1] Collister D, Ferguson T, Komenda P, Tangri N (2016). The patterns, risk factors, and prediction of progression in chronic kidney disease: A narrative review. Semin. Nephrol..

[2] Kakani E, Elyamny M, Ayach T, El-Husseini A (2019). Pathogenesis and management of vascular calcification in CKD and dialysis patients. Semin. Dial..

[3] Zhang L, Yao L, Bian WJ, Rui HL (2009). Severe uremic leontiasis ossea ameliorated by total parathyroidectomy. Kidney Int..

[4] Sagliker Y (2004). Sagliker syndrome: Uglifying human face appearance in late and severe secondary hyperparathyroidism in chronic renal failure. Semin. Nephrol..

[5] Proctor R, Kumar N, Stein A, Moles D, Porter S (2005). Oral and dental aspects of chronic renal failure. J. Dental Res..

[6] Raubenheimer E, Noffke C, Mohamed A (2015). Expansive jaw lesions in chronic kidney disease: Review of the literature and a report of two cases. Oral Surg., Oral Med., Oral Pathol., Oral Radiol..

[7] Allen MR, Chen NX, Gattone VH, Moe SM (2013). Adverse mandibular bone effects associated with kidney disease are only partially corrected with bisphosphonate and/or calcium treatment. Am. J. Nephrol..

[8] Lee MM (2010). Characterization of mandibular bone in a mouse model of chronic kidney disease. J. Periodontol..

[9] Watanabe K (2018). Newly developed rat model of chronic kidney disease-mineral bone disorder. J. Atheroscler. Thromb..

[10] Moe S (2006). Definition, evaluation, and classification of renal osteodystrophy: A position statement from Kidney Disease: Improving global outcomes (KDIGO). Kidney Int..

[11] Varela A, Jolette J (2018). Bone toolbox: Biomarkers, imaging tools, biomechanics, and histomorphometry. Toxicol. Pathol..

[12] Morgan EF, Unnikrisnan GU, Hussein AI (2018). Bone mechanical properties in healthy and diseased states. Annu. Rev. Biomed. Eng..

[13] Meng Y, Zhang H, Li Y, Li Q, Zuo L (2014). Effects of unfractionated heparin on renal osteodystrophy and vascular calcification in chronic kidney disease rats. Bone.

[14] Meng Y (2018). A comparative study on e ffects of low molecular weight heparin and unfractionated heparin on renalosteodystrophy and vascular calcification in rats with chronic kidney disease. Chin. J. Osteoporos..

[15] Reeves PG, Nielsen FH, Fahey GC (1993). AIN-93 purified diets for laboratory rodents: Final report of the American institute of nutrition ad hoc writing committee on the reformulation of the AIN-76A rodent diet. J. Nutr..

[16] Castro BBA (2020). Digital radiography as an alternative method in the evaluation of bone density in uremic rats. J. Braz. Nefrol..

[17] Liu X (2019). Huangqi-danshen decoction ameliorates adenine-induced chronic kidney disease by modulating mitochondrial dynamics. Evid. Based Complement Altern. Med..

[18] Aido M (2014). Relationship between nanoscale mineral properties and calcein labeling in mineralizing bone surfaces. Connect Tissue Res..

[19] Fujihara R (2018). Histomorphometry of ectopic mineralization using undecalcified frozen bone sections. Microsc. Res. Tech..

[20] de Bakker CM (2015). muCT-based, in vivo dynamic bone histomorphometry allows 3D evaluation of the early responses of bone resorption and formation to PTH and alendronate combination therapy. Bone.

[21] Eratalay YK (1981). Bone growth in the rat mandible following every-day or alternate-day methylprednisolone treatment schedules. Arch. Oral Biol..

[22] Sato M, Zeng GQ, Turner CH (1997). Biosynthetic human parathyroid hormone (1–34) effects on bone quality in aged ovariectomized rats. Endocrinology.

[23] van Eijden TM (2000). Biomechanics of the mandible. Crit. Rev. Oral Biol. Med..

[24] Bozzini C, Picasso E, Champin G, Bozzini CE, Alippi RM (2015). Effect of physical consistency of food on the biomechanical behaviour of the mandible in the growing rat. Eur. J. Oral Sci..

[25] Fujita Y, Watanabe K, Uchikanbori S, Maki K (2011). Effects of risedronate on cortical and trabecular bone of the mandible in glucocorticoid-treated growing rats. Am. J. Orthod. Dentofac. Orthop..

[26] Parfitt AM (1987). Bone histomorphometry: Standardization of nomenclature, symbols, and units. Report of the ASBMR histomorphometry nomenclature committee. J. Bone Miner. Res..

[27] Isakova T (2011). Fibroblast growth factor 23 is elevated before parathyroid hormone and phosphate in chronic kidney disease. Kidney Int..

[28] Raubenheimer EJ, Noffke CE, Hendrik HD (2015). Chronic kidney disease-mineral bone disorder: An update on the pathology and cranial manifestations. J. Oral Pathol. Med..

[29] Lopes ML (2015). Severe maxillofacial renal osteodystrophy in two patients with chronic kidney disease. Oral Maxillofac. Surg..

[30] Pontes FSC (2018). Oral and maxillofacial manifestations of chronic kidney disease-mineral and bone disorder: A multicenter retrospective study. Oral Surg., Oral Med., Oral Pathol., Oral Radiol..

[31] Okada H, Davies JE, Yamamoto H (2000). Brown tumor of the maxilla in a patient with secondary hyperparathyroidism: A case study involving immunohistochemistry and electron microscopy. J. Oral. Maxillofac. Surg..

[32] Adachi PL, da Silva Santos PS, de Magalhaes MH, Martins MT (2007). Renal osteodystrophy manifesting as localized enlargement of the jaw. Nephrol. Dial. Transplant..

[33] Norrdin RW (1975). Fibrous osteodystrophy with facial hyperostosis in a dog with renal cortical hypoplasia. Cornell. Vet..

[34] Beaupied H, Lespessailles E, Benhamou CL (2007). Evaluation of macrostructural bone biomechanics. Joint Bone Spine.

[35] Ni LH (2018). A rat model of SHPT with bone abnormalities in CKD induced by adenine and a high phosphorus diet. Biochem. Biophys. Res. Commun..

[36] Meng Y (2018). Mineral and bone metabolism disorder in an adenine-induced rat model of chronic kidney disease. Chin. J. Comp. Med..

[37] Chen J (2015). The cortical bone mass and structure in a mouse model of renal osteodystrophy. J. Nephrol. Dialy Transplant..

[38] Swallow EA (2019). Skeletal levels of bisphosphonate in the setting of chronic kidney disease are independent of remodeling rate and lower with fractionated dosing. Bone.

[39] Li L, Li A, Gan L, Zuo L (2023). Roxadustat improves renal osteodystrophy by dual regulation of bone remodeling. Endocrine.

[40] Yamashita S (2023). Chronic kidney disease compromises structural and mechanical properties of maxillary cortical bone in a rat model. J. Prosthodont. Res..

[41] Calciolari E, Donos N, Park JC, Petrie A, Mardas N (2016). A systematic review on the correlation between skeletal and jawbone mineral density in osteoporotic subjects. Clin. Oral Implants Res..

